# Neuromuscular activation of the quadriceps femoris, including the vastus intermedius, during isokinetic knee extensions

**DOI:** 10.1038/s41598-023-34532-x

**Published:** 2023-05-11

**Authors:** Hiroshi Akima, Kazuhiro Maeda, Norihiro Shima

**Affiliations:** 1 Physical Fitness and Sports, Research Center of Health, Nagoya University, 1 Furo, Chikusa, Nagoya, Aichi 464-8601 Japan; 2 Graduate School of Education and Human Development, Nagoya University, 1 Furo, Chikusa, Nagoya, Aichi 464-8601 Japan; 3 School of Sports and Health Science, Tokai Gakuen University, 21-233 Nishi-no-hora, Ukigai, Miyoshi, Aichi 470-0207 Japan

**Keywords:** Bone quality and biomechanics, Biomedical engineering

## Abstract

The purpose of this study was to compare the neuromuscular activation patterns of the individual muscles of the quadriceps femoris (QF), including the vastus intermedius (VI), during isokinetic concentric (CON) and eccentric (ECC) contractions. Thirteen healthy men performed maximum isokinetic CON and ECC knee extensions at angular velocities of 30, 90, and 120°/sec at knee joint angles from 80 to 180° (180° = full extension). The surface electromyographic (EMG) activities of the four individual muscles of the QF were recorded. The root mean squares of the EMG signals were normalized by the root mean square (nRMS) during CON contraction at 30°/sec. To investigate the nRMS changes, we classified the range of motion into four subcategories for each CON and ECC contraction. The nRMS of the VI was significantly higher in the flexed position during CON and ECC contractions at all velocities, and gradually decreased toward the extended positions regardless of the type of muscle contraction or angular velocity. These results suggest that the QF undergoes neuromuscular activation in a joint angle-dependent manner. In particular, the VI may contribute greatly during flexed contractions, independent of the type of contraction and angular velocity.

## Introduction

The quadriceps femoris (QF) is an important muscle group that plays crucial functional roles in human daily life movements and physical activities. For example, the QF contributes to human movements such as walking, running, climbing stairs, and cycling. The QF group are also strong antigravity muscles that constantly support the human body during daily life. Therefore, the QF is sensitive to the effects of gravity and will quickly atrophy due to disuse^1–3^. Conversely, an increase in activity induces, muscle hypertrophy^4,5^.

The QF is composed of three superficial muscles (rectus femoris (RF), vastus lateralis (VL), and vastus medialis (VM)], and one deep muscle [vastus intermedius (VI)). These four muscles may have specific functions during knee extension. The normalized root mean square (nRMS) of the surface electromyographic (EMG) activity of the VM and VI significantly decreases during isometric knee extension force when the knee joint angle changes from 115 to 165° (180° = fully extended)^6^. Furthermore, during isotonic contractions, the nRMS of the VI is significantly higher than that of the VL, VM, and RF at 60% to 100% of the one-repetition maximum (1RM) during the extension (i.e., concentric) and flexion (i.e., eccentric) phases with a flexed knee^7^. These previous studies suggest that the VI may make a specific contribution at the flexed position of knee extension; however, it is unclear whether this activation pattern of the VI is specific to isometric or isotonic contractions.

In addition to isometric and isotonic contractions, numerous studies have evaluated isokinetic muscle contraction^8,9^. However, the details of the neuromuscular activity of the individual muscles of the QF during isokinetic muscle exertion have not been clarified. Isokinetic muscle action is unique because, the effect of inertia is eliminated during muscle exertion, especially at low angular velocities. Many studies have used the VL alone or the superficial muscles of the QF to evaluate the neuromuscular activation pattern of the QF^10–13^; however, it is possible that the full neuromuscular function of the QF during muscle contractions cannot be captured by evaluating the VL alone or only the superficial muscles of the QF^6,7,14^.

We previously assessed the validity of recording the neuromuscular activation pattern of the VI by combining surface and intramuscular EMG recordings during isometric contractions^6,15–17^ and dynamic contractions^7,18^ at different joint angles. These previous studies revealed that the EMG signal detected from the VI seems to have negligible cross-talk from adjacent muscles^16^. The root mean square (RMS) detected from a surface electrode shows similar changes to the RMS from an intramuscular electrode when the joint angle changes^6^.

The purpose of the present study was to compare the nRMS patterns of the VI and three other synergist muscles of the QF during isokinetic concentric and eccentric knee extension performed with maximal effort. We hypothesized that the nRMS patterns of the VI would be different to the nRMS patterns of the other three QF muscles during both concentric and eccentric contractions at various angular velocities.

## Materials and methods

### Participants

Thirteen healthy men participated in this experiment. Prior to the experiment, the study protocol, purposes, risks, and benefits were explained to all participants, and written informed consent was obtained. The mean (± standard error of the mean) age was 20.8 ± 0.7 years, height was 172.1 ± 6.3 cm, and weight was 64.8 ± 8.0 kg. All experimental protocols were approved by the Ethics Committee of the Research Center of Health, Physical Fitness & Sports, Nagoya University (#19-05) and were in accordance with the guidelines outlined in the Declaration of Helsinki.

### Experimental protocol

All participants practiced performing isokinetic concentric and eccentric knee extension tasks with maximal effort 1 week prior to the initial experiment. The participants came back to the laboratory after 1 week of practice session to perform maximum isokinetic concentric and eccentric knee extensions at 30, 90, and 120°/sec in random order. The surface EMG signals of the QF were also recorded during the task.

### Isokinetic concentric and eccentric knee extension tasks

The participants performed maximum isokinetic concentric and eccentric knee extensions at angular velocities of 30, 90, and 120°/sec using an isokinetic dynamometer (CON-TREX MJ, CMV AG, Dubendorf, Switzerland). The participants sat on the equipment chair with their hips at angles of approximately 100° (180° = full extension) and knees at 80° (180° = full extension) in the resting position. The knee padding length was adjusted to the length of the lower limb and was kept constant during the task. The participants performed several repetitions of isokinetic knee extension trials with submaximal and maximal efforts as a warm-up exercise.

Isokinetic concentric and eccentric peak torque values were measured at 30, 90, and 120°/sec during maximum knee extension with an 80° to 180° range of motion (ROM). The muscle contraction type (i.e., concentric or eccentric) and angular velocity were randomly chosen for each participant. The participants performed two trials at 30°/sec and three trials at 90 and 120°/sec with maximal effort, with at least 1 min between trials. Knee joint angle signals were measured by an electrogoniometer (SG150, Biometrics, Ltd., Gwent, UK) and sampled at 2 kHz using an AC/DC converter (PowerLab 16SP; ADInstruments, Melbourne, Victoria, Australia). The signals were collected onto a personal computer (MacMini; Apple Inc., Cupertino, CA., USA) with Chart software (LabChart v.8.1.17; ADInstruments).

### EMG recording

Surface EMG signals from the VI, VL, VM, and RF were recorded with active electrodes during the isokinetic knee extension task, as previously described^7,18^. EMG signals were measured using a single-differential, 4.1 × 2.0 × 0.5-cm electrode with a 1-cm interelectrode distance, an input impedance of > 10^15^ Ω/0.2 pF, and a 90 dB common mode rejection ratio. The sensor amplifier units were set at a gain of 1000-fold with a frequency response of 20 to 450 Hz (DE-2.1 sensor; Main Amplifier Unit: Bagnoli-8, Delsys, Boston, MA, USA). Signals from the EMG system were sampled at 2000 Hz using the abovementioned A/D converter and personal computer to synchronize the signal with the torque and knee joint angle^7,18^.

The skin was shaved, abraded, and cleaned with alcohol prior to attaching the electrodes. The electrodes were placed at the mid-point between the head of the greater trochanter and the inferior edge of the patella for the VL, at the mid-point of the line joining the anterior superior iliac spine and the superior patellar pole for the RF, and slightly proximal and medial to the patella for the VM according to our previous study^7,18^. All electrodes were placed parallel to the estimated fiber direction.

We assessed the VI using a procedure similar to that used in our previous studies^7,15,18^. Briefly, we previously used magnetic resonance imaging and ultrasonography to confirm the location of the superficial region of the VI at the lateral-distal portion of the thigh in 45 healthy men^16^. For all participants, we used different colored marker pens to trace the edges of the superficial regions of the VI at knee joint angles of 80° and 160° using ultrasonography (ProSound SSD-a7; ALOKA CO., LTD., Tokyo, Japan). Then, we identified the common region of the VI on the skin surface between the two knee joint angles. Ultrasonography was used to determine electrode placement within the common region, while noting volume conductance^16^. We demonstrated that the EMG activity of the VI has a negligible amount of cross-talk with the adjacent VL^16^ and that the surface EMG signals of the VI are closely associated with the needle EMG signal of the VI (*r* = 0.926–0.991)^6^. The electrodes for the VI were placed parallel to the estimated longitudinal axis of the muscle. The reference electrode was attached to the iliac crest.

### Data analysis

To investigate how the RMS changes with knee joint angle changes during isokinetic contractions, we classified the ROM into four subcategories for each concentric and eccentric contraction in accordance with the knee joint angle determined by the isokinetic dynamometer. These subcategories were: joint angle from 80 to 100° (CON80–100), 100° to 120° (CON100–120), 120° to 140° (CON120–140), and 140° to 160° (CON140–160) during concentric contractions, and joint angle from 160 to 140° (ECC160–140), 140° to 120° (ECC140–120), 120° to 100° (ECC120–100), and 100° to 80° (ECC100–80) during eccentric contractions. The RMS values of the four muscles and mean torque were calculated for each subcategory. To normalize the EMG signals, the RMS signals of subcategories during isokinetic concentric and eccentric contractions at all angular velocities were normalized by the RMS (nRMS), during concentric contraction at 30°/sec at knee joint angles from 80° to 160°. The RMS was calculated using a previously described Eq. ^19^.

### Statistical analysis

All data are presented as the mean and standard error of the mean. A two-way (ROM × muscle) ANOVA with repeated measures was also used to compare the nRMS across timepoints and muscles for each concentric contractions or eccentric contractions. In the case of a two-factor interaction or main effects, a Tukey’s post hoc test was used to identify significant differences among the four muscles for each ROM. A Mauchly sphericity test was applied; if the sphericity assumption was violated, the Greenhouse–Geisser correction factor was used to control for Type I errors. The level of significance was set at *P* < 0.05. Statistical analysis was performed using IBM SPSS statistics software (version 27.0, IBM, Tokyo, Japan).

## Results

**Figure 1:**
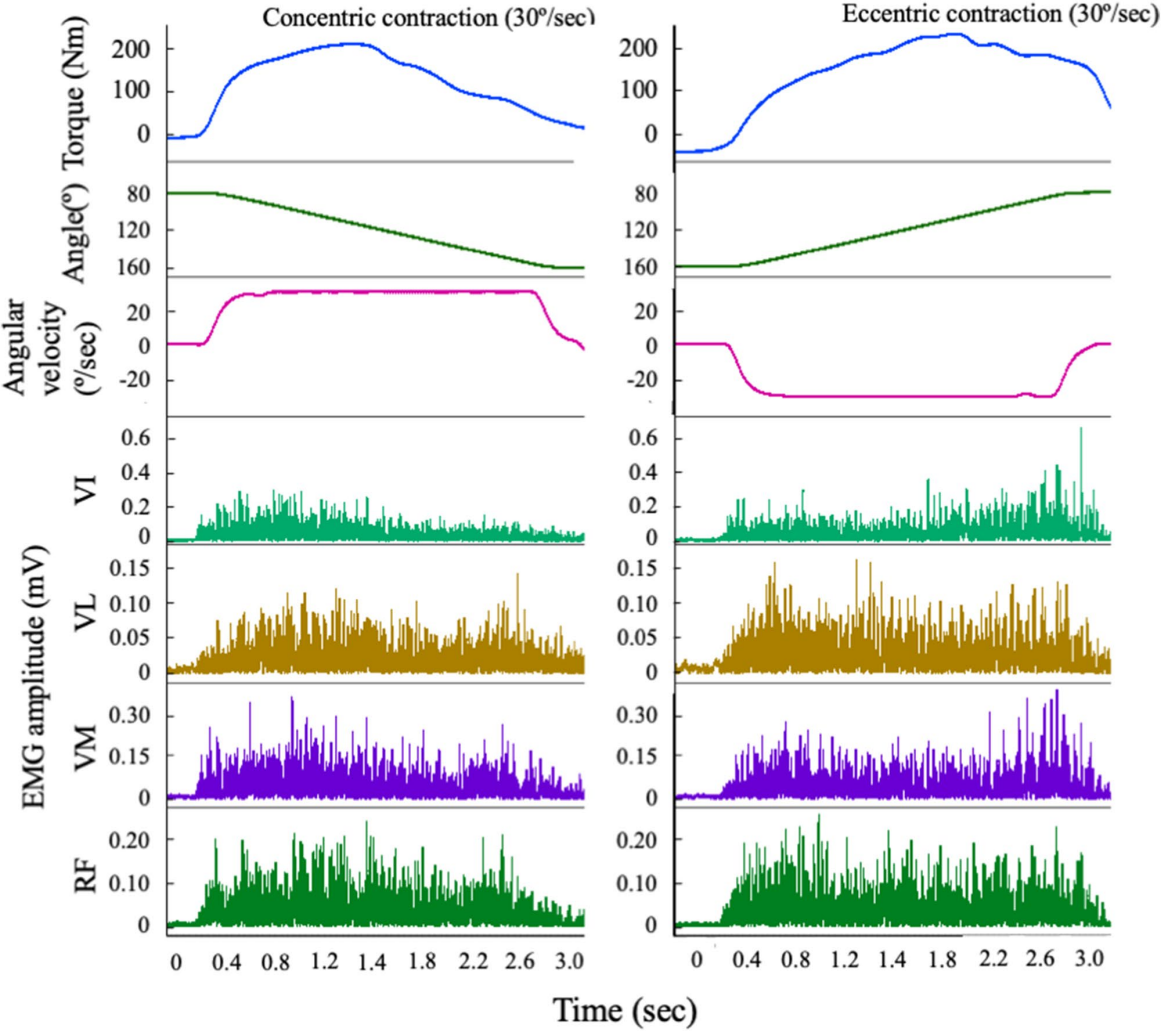
Representative data of torque, knee joint angle, angular velocity, and the EMG amplitude of the vastus intermedius, vastus lateralis, vastus medialis, and rectus femoris during concentric and eccentric isokinetic knee extensions at 30, 90, and 120°/sec.

Figure 1 shows representative data of torque, knee joint angle, angular velocity, and the EMG amplitude of the VI, VL, VM, and RF during concentric and eccentric isokinetic knee extensions at 30°/sec.

### Torque-knee joint angle relationships

**Figure 2:**
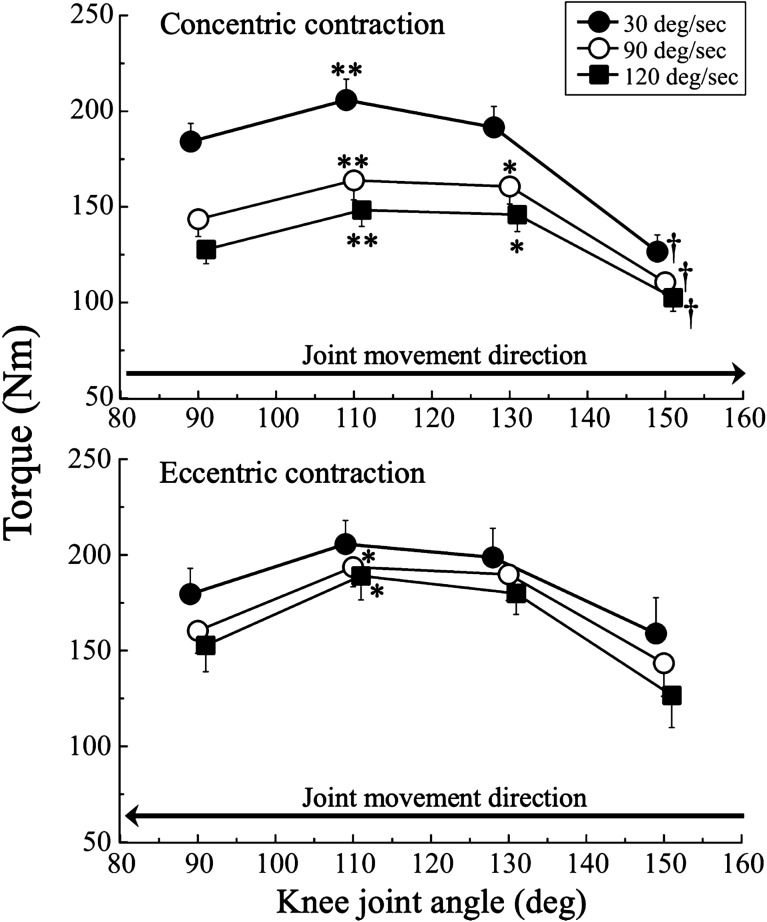
Torque-knee joint angle relationships during concentric and eccentric isokinetic knee extensions at 30, 90, and 120°/sec *; P < 0.05, **; *P* < 0.01, †; *P* < 0.001 versus knee joint angle of 90 degree.

Figure 2 shows the torque-knee joint angle relationships during concentric and eccentric isokinetic knee extensions at 30, 90, and 120°/sec. Two-way ANOVA revealed significant ROM effects at 30°/sec (F_3.0,69.0_ = 23.1, *P* = 0.001, η^2^ = 0.501), 90°/sec (F_3.0,69.0_ = 23.1, *P* = 0.001, η^2^ = 0.501), and 120°/sec (F_3.0,69.0_ = 32.3, *P* = 0.001, η^2^ = 0.584), but there were no significant angular velocity effect and ROM-by-angular velocity interactions. During concentric contractions, one-way ANOVA showed that the ROM significantly affected the torque at 30°/sec (F_1.6,19.6_ = 67.2, *P* = 0.001, η^2^ = 0.848), 90°/sec (F_1.3,16.1_ = 39.6, *P* = 0.001, η^2^ = 0.768), and 120°/sec (F_1.6,19.6_ = 67.2, *P* = 0.001, η^2^ = 0.848). A pairwise comparison revealed that the torque values during CON80–100 at 30°/sec were significantly lower than those during CON100–120 (*P* = 0.003), but were significantly higher than those during CON140–160 (*P* = 0.001). The torque values during CON80–100 at 90 and 120°/sec were significantly lower than those during CON100–120 (both *P* = 0.002) and CON120–140 (both *P* = 0.010) for each angular velocity, but were significantly higher than those during CON140–160 (both *P* = 0.001).

During eccentric contractions, one-way ANOVA showed that the ROM significantly affected the torque at 90°/sec (F_1.1,12.3_ = 6.4, *P* = 0.023, η^2^ = 0.369) and 120°/sec (F_1.2,13.1_ = 7.3, *P* = 0.015, η^2^ = 0.400). A pairwise comparison revealed that the torque during ECC100–80 was significantly lower than that during ECC120–100 (all *P* = 0.048) at 90 and 120°/sec for each angular velocity.

### nRMS-knee joint angle relationships during concentric contractions

**Figure 3:**
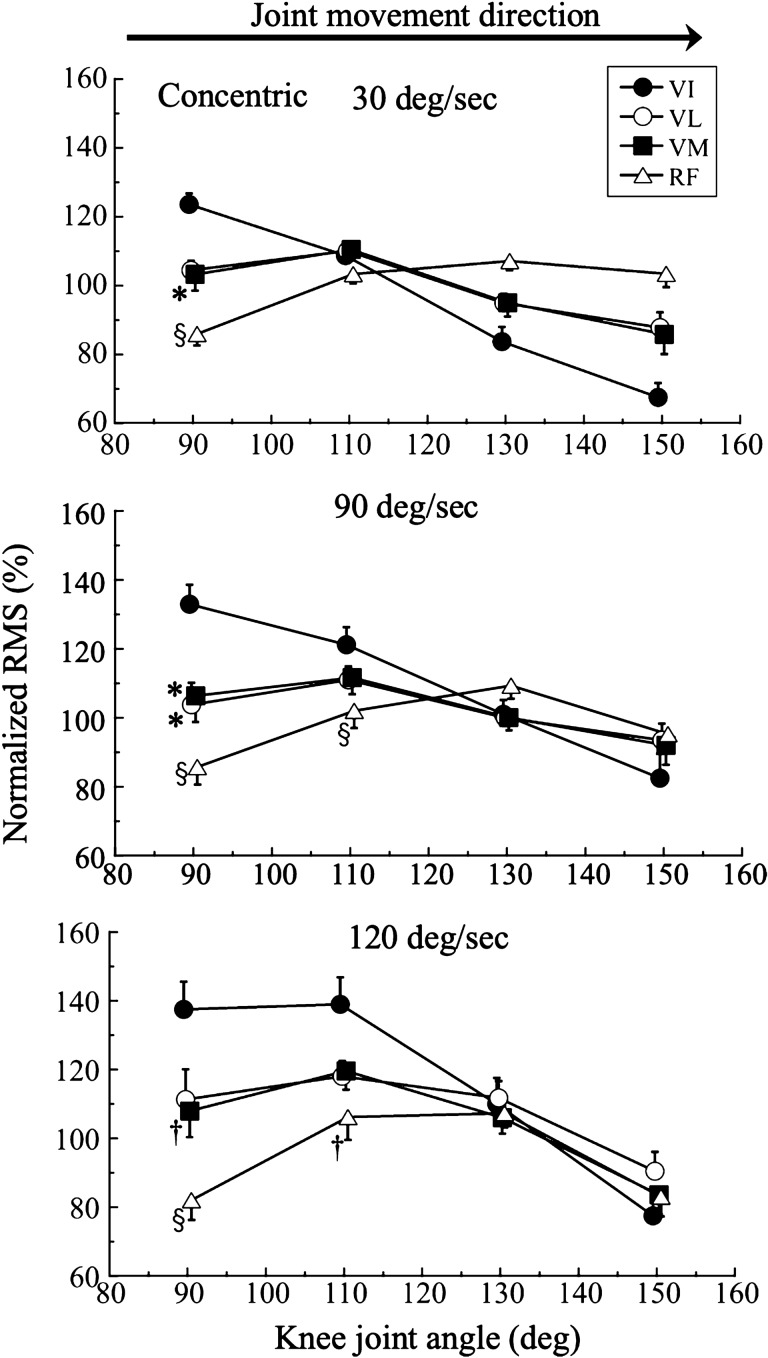
Normalized root mean square (RMS) during concentric isokinetic knee extensions at 30, 90, and 120°/sec Muscle comparison: *; *P* < 0.05, †; *P* < 0.01, §; *P* < 0.001 versus vastus intermedius at the same knee joint angle. VI, vastus intermedius, VL, vastus lateralis, VM, vastus medialis, RF, rectus femoris.

Figure 3 shows the nRMS-knee joint angle relationships during isokinetic concentric contractions of knee extensions at 30, 90, and 120°/sec. Two-way ANOVA revealed significant muscle effects at 30°/sec (F_3.0,48.0_ = 8.6, *P* = 0.001, η^2^ = 0.351) and 120°/sec (F_3.0,48.0_ = 3.8, *P* = 0.017, η^2^ = 0.190), and significant ROM effects at 30°/sec (F_1.8,87.1_ = 22.7, *P* = 0.001, η^2^ = 0.322), 90°/sec (F_1.9,90.2_ = 15.7, *P* = 0.001, η^2^ = 0.246), and 120°/sec (F_2.0,95.1_ = 34.8, *P* = 0.001, η^2^ = 0.420). We also found muscle-by-ROM interactions at 30°/sec (F_5.4,87.1_ = 10.7, *P* = 0.001, η^2^ = 0.400), 90°/sec (F_5.6,90.2_ = 6.7, *P* = 0.001, η^2^ = 0.295), and 120°/sec (F_5.9,95.1_ = 4.9, *P* = 0.001, η^2^ = 0.236).

A pairwise muscle comparison showed that the nRMS of the VI during CON80–100 was significantly higher than that of the VM (*P* = 0.039) and RF (*P* = 0.001) at 30°/sec, the VL (*P* = 0.010), VM (*P* = 0.023), and RF (*P* = 0.001) at 90°/sec, and the VM (*P* = 0.039) and RF (*P* = 0.001) at 120°/sec. The nRMS of the VI during ROM CON100–120 was also significantly higher than that of the RF (*P* = 0.003 to 0.029) at 30, 90, and 120°/sec.

### nRMS-knee joint angle relationships during eccentric contractions

**Figure 4:**
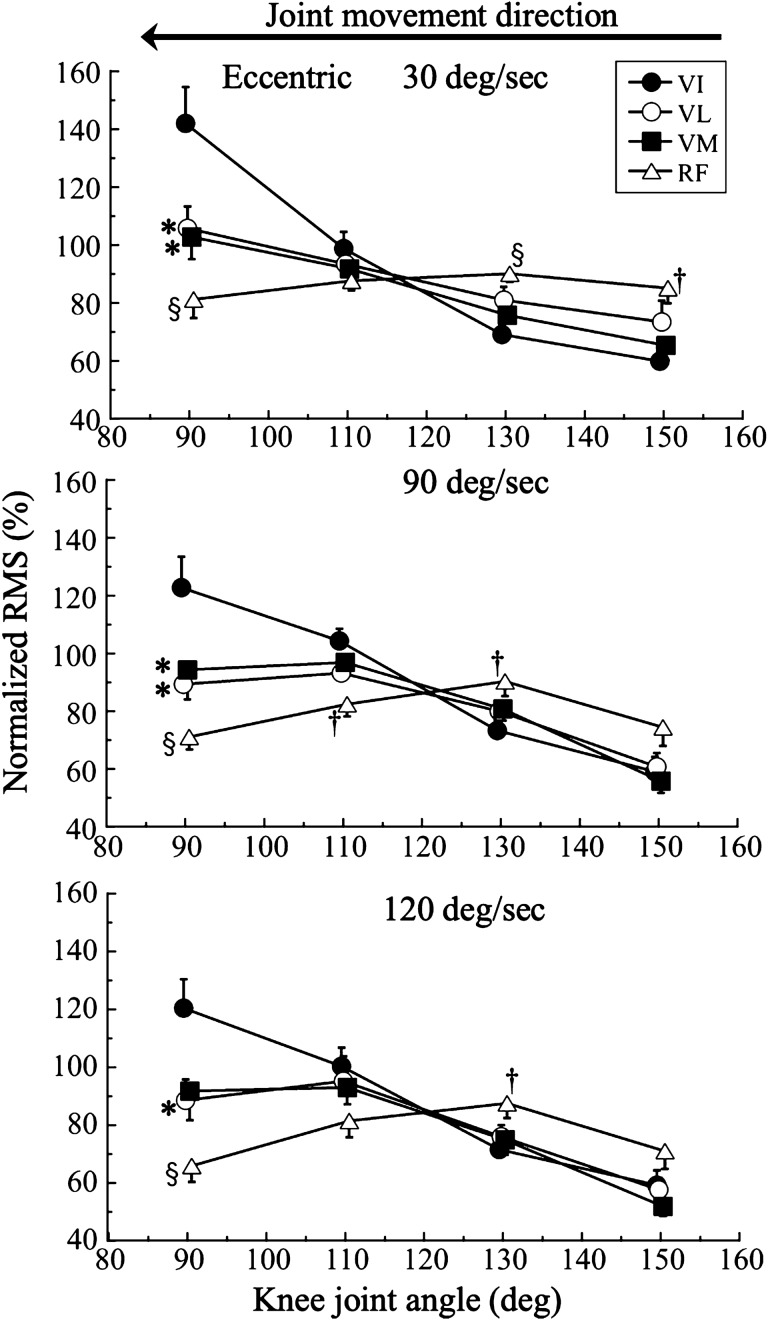
Normalized root mean square (RMS) during eccentric isokinetic knee extensions at 30, 90, and 120°/sec Muscle comparison: *; *P* < 0.05, †; *P* < 0.01, §; *P* < 0.001 versus vastus intermedius at the same knee joint angle. VI, vastus intermedius, VL, vastus lateralis, VM, vastus medialis, RF, rectus femoris.

Figure 4 shows the nRMS-knee joint angle relationships during isokinetic eccentric contractions of knee extensions at 30, 90, and 120°/sec. Two-way ANOVA revealed no significant muscle effects. However, we found significant ROM effects at 30°/sec (F_1.6,71.1_ = 39.6, *P* = 0.001, η^2^ = 0.473), 90°/sec (F_1.7,73.1_ = 41.1, *P* = 0.001, η^2^ = 0.483), and 120°/sec (F_1.9,85.7_ = 35.0, *P* = 0.001, η^2^ = 0.443). We also found muscle-by-ROM interactions at 30°/sec (F_4.8,77.1_ = 10.0, *P* = 0.001, η^2^ = 0.406), 90°/sec (F_5.0,73.1_ = 8.7, *P* = 0.001, η^2^ = 0.372), and 120°/sec (F_5.8,85.7_ = 7.1, *P* = 0.001, η^2^ = 0.327).

A pairwise comparison among muscles revealed that the nRMS of the VI was significantly lower than that of the RF during ECC160–140 (*P* = 0.008) and ECC140–120 (*P* = 0.001) at 30°/sec and during ECC140–120 at 90°/sec. In contrast, the nRMS of the VI was significantly higher than that of the VL (*P* = 0.022), VM (*P* = 0.012), and RF (*P* = 0.001) during ECC100–80 at 30°/sec, significantly higher than that of the RF (*P* = 0.002) during ECC120–100, significantly higher than that of the VL (*P* = 0.015), VM (*P* = 0.043), and RF (*P* = 0.001) during ECC100–80 at 90°/sec, and significantly higher than that of the VL (*P* = 0.029) and RF (*P* = 0.001) during ECC100–80 at 120°/sec.

## Discussion

Among the individual QF muscles, the VI has unique morphological and functional characteristics^6,7^. The VI is located in the deep region of the QF, and its fascicles directly attach to the femur over a wide area^14^. Due to these specific morphological features, the fascicle behavior of the VI during contraction is different to that of the other QF muscles^20,21^. A previous comparison of the nRMS of the VI and VL during isometric contraction at knee joint angles of 90° (= baseline), 115°, 140°, and 165° showed that the nRMS of the VI is 60% and 40% of the baseline value at joint angles of 140° and 165°, respectively, which is significantly lower than that of the VL and RF at the same joint angles^6^. Another study of isotonic contractions showed that the nRMS of the VI is significantly higher in the flexed position, but is significantly lower in the extended position when the load is heavier than 60% of the 1RM^7^. These two studies clearly show that the activation characteristics of the VI are dependent on the knee joint angle; however, it is unknown whether this activation pattern is specific for only isometric and isotonic contractions. No study has shown the activation patterns of the VI during “isokinetic” knee extensions. In the present study, we examined whether the neuromuscular activation of the VI was higher at flexed knee joint angles during isokinetic concentric and eccentric contractions with maximal effort.

Our assessment of the torque-knee joint angle relationship during concentric contractions showed that the torque significantly increased once at a knee joint angle of 110°, then significantly decreased during CON120–140 at 30°/sec and during CON140–160 at 90 and 120°/sec with knee extension. A previous study reported that the peak torque appears when the knee shifts to the flexed position with increasing angular velocities during isokinetic knee extensions^22^. A similar result was found in the present study (Fig. 2). The peak torque was found during CON100–120 at 30°/sec; however, the peak torque occurred between CON100–120 and CON120–140 at 90 and 120°/sec. This shows that the peak torque shifted to the extended position with increasing angular velocity.

During eccentric contractions, the torque-knee joint angle relationships were similar across angular velocities in the present study. Overall, there were no significant differences among the ROM subcategories across angular velocities. However, the torque values were significantly higher during ECC120–100 at 90 and 120°/sec compared with ECC100–80. To the best of our knowledge, no previous studies have performed similar comparisons of eccentric contractions.

The present study showed that, compared with the other QF muscles, the VI had the highest nRMS at the knee flexed position during isokinetic concentric and eccentric contractions (Figs. 3 and 4). This suggests that the VI has a specific neuromuscular characteristic that causes a relatively higher activity than the other QF muscles at the flexed position during isokinetic contraction. This angle-specific higher activation of the VI was independent of the type of muscle contraction, occurring during isokinetic contractions (current study), isometric contraction^6^, and isotonic contraction^7^, and the direction of knee joint rotation^7^. According to the findings of the present study and previous studies, this neuromuscular activation pattern of the VI reflects its intrinsic neuromuscular system properties.

The nRMS of the VI decreased linearly with the knee joint angle during concentric and eccentric contractions at almost all angular velocities. This nRMS changing pattern with knee joint angle changes was unique in the VI compared with the other individual QF muscles. Previous studies have found a similar angle-associated pattern during isotonic knee extension at loads higher than 60% of the 1RM^6,7^ and during maximal isometric contractions at various knee joint angles^6^. According to these previous studies, the nRMS of the VI has a unique activation characteristic, and this knee joint angle-specific activation pattern is independent of the type and direction of muscle contractions. We considered possible explanations for the unique activation pattern of the VI, especially at knee joint flexed. First, this type of activation pattern may reflect the intrinsic characteristics of the VI. Surface and intramuscular EMG techniques have shown that the nRMS of the VI synchronously decreases with knee extension^6^, indicating that the nRMS of the VI is sensitive to changes in the knee joint angle. Second, the VI is the most efficient of the QF muscles when performing knee extension using amputated limbs^14^. According to Lieb et al.^14^, they showed that the VI is the most efficient muscle with the least amount of weight individual muscles to carry out knee extension of the QF based on amputated limb experiments. This higher efficiency of the VI may be seen when the knee is in the flexed position in this study independent of types of muscle contraction. As we have discussed, the neuromuscular function of VI seems to be unique compared to other muscles. As we discussed, the neuromuscular function of VI seems to be unique compared to the other QF muscles, but quantitative comparisons such as M waves have not been made on a muscle-by-muscle basis. In other words, the comparison of muscle nRMS in this study is a qualitative. Therefore, that is a point to be discussed in the future studies, and the interpretation of the results of this study should be done with caution.

The shape of the nRMS-knee joint angle relationship during eccentric contractions at all tested angular velocities seemed to be similar to that during concentric contractions, even when the knee joint was moving in the opposite direction in each type of muscle contraction. The nRMS was significantly higher in the VI than the other QF muscles when the knee was in the flexed position; however, this higher nRMS disappeared with extension of the knee, independent of the type of contraction and angular velocity. These results suggest that the similar nRMS-knee joint angle relationship seen in concentric and eccentric contractions may be associated with the fine regulation by the nervous system and/or architectural properties of the QF.

There was a muscle-specific shape of the nRMS-knee joint angle relationship for each individual muscle of the QF for each angular velocity. The nRMS-knee joint angle relationship of the VI appeared as a descending right relationship during concentric and eccentric contractions at almost all angular velocities; in contrast, the nRMS-knee joint angle relationships of the VL, VM, and RF had a slightly mountainous shape, irrespective of the type of contraction and angular velocity. There are a few possible explanations for the specific nRMS-knee joint angle relationships of the VI and the other QF muscles. First, these relationships may be affected by differences in anatomical features between the VI and the VL, VM, or RF. The VI is reported the most efficient of the QF muscles during knee extension of amputated limbs^14^, and the VI may have a specific function in according with its morphological and architectural features on B-mode ultrasonography^21^. Second, the architecture of the VI is a better predictor of knee extension force. A previous study showed that the muscle thickness and pennation angle of the VI explain 91% of the isometric knee extension force at a knee joint angle of 90°^20^, which is consistent with the current results.

A novel finding of the present study was that the patterns of change in the nRMS of the VL and VM with changes in the knee joint angle were very well matched, independent of the type of muscle contraction and angular velocity. This result suggests that the VL and VM are close synergistic muscles that perform isokinetic knee extension tasks. Similarly, a previous study found a significant moderate correlation between the spin–spin (T2) relaxation times (which mainly reflect muscle activation) of the VL and VM (r = 0.347, *P* < 0.01) based on muscle functional magnetic resonance imaging after repetitive isokinetic knee exercises^23^. In contrast, the nRMS-knee joint angle relationship of the VI was in the opposite direction to that of the RF under all conditions (Figs. 3 and 4). The nRMS of the VI decreased with knee extension, while that of the RF increased once and then slightly decreased as the knee moved toward the extended position. These results suggest that the activation levels of the VL and VM may be almost maximal throughout the ROM of the knee joint. However, the VI showed a gradual decrease in its muscle activation from knee flexion to extension, while the RF showed a slight increase in muscle activity in knee extension. These muscle-specific activation patterns may be related to force–length relationships, motor unit firing patterns, or other neuromuscular and physiological factors. In addition, the behavior of the VM during isokinetic muscle force has not been clarified at all, although the VL has been used in many studies. The current study is the first to reveal neuromuscular characteristics of the QF muscle other than the VL during isokinetic concentric and eccentric contraction. Isokinetic testing is often used in orthopedic rehabilitation and sports performance. The greatest strength of this study is that it evaluated neuromuscular functions other than VL during isokinetic knee extension exercise.

In this study, we found some limitation in this study. First, the methods for EMG normalization may affect the findings. As we used dynamic isokinetic knee extension exercises, this type of muscle contraction may affect the EMG normalization. Therefore, we may need to be very careful when interpreting the results. Second, the number of participants were relatively smaller, which may induce type II error. Therefore, the data obtained should be looked at carefully. Third, normally, the muscle force during eccentric contraction torque is higher than the concentric contraction. However, no difference was found between concentric and eccentric contractions in this study. Participants practiced muscle exertion 1 week prior to the main experiment as shown in Materials and Methods. The reasons for the lack of typical muscle torque data could not be determined in this study. It will be necessary to clarify the cause of the problem in our future study.

In conclusion, we characterized the neuromuscular activation of the four individual QF muscles during concentric and eccentric isokinetic knee extensions. The neuromuscular activation pattern of the VI during isokinetic contraction was significantly higher in knee flexion and lower in knee extension. This activation pattern was independent of the type of contraction and angular velocity. The nRMS-knee joint angle relationships in the VL and VM were consistent across all contraction types and angular velocities. These results suggest that the individual QF muscles change their activity in a knee joint angle-dependent manner or in contribution with other synergistic muscles.

## Data Availability

The datasets generated during and/or analyzed during the current study are available from corresponding author on reasonable request.
